# Serotonin Syndrome after Sertraline Overdose in a Child: A Case Report

**DOI:** 10.1155/2013/897902

**Published:** 2013-12-19

**Authors:** Joana Grenha, Ana Garrido, Hernani Brito, Maria José Oliveira, Fátima Santos

**Affiliations:** ^1^Department of Pediatrics, Centro Hospitalar de Vila Nova de Gaia e Espinho, EPE, Rua Doutor Francisco Sá Carneiro 4400-129 Vila Nova de Gaia, Portugal; ^2^Department of Pediatrics, Centro Hospitalar do Médio Ave, EPE, Largo Domingos Moreira 4780-371 Santo Tirso, Portugal; ^3^Pediatric Intensive Care Unit, Centro Hospitalar de São João, EPE, Alameda Prof. Hernâni Monteiro, 4200-319 Porto, Portugal

## Abstract

Serotonin syndrome is a potentially life-threatening drug effect. It may be misdiagnosed because it has mostly been reported in adults. *Case Report*. An 8-year-old girl with behavioral problems and medicated with risperidone and sertraline was admitted in the emergency department after she had taken voluntarily 1500 mg of sertraline (50 mg/kg). At admission, she had marked agitation, visual hallucinations, diaphoresis, flushing, and tremor. She had fever and periods of hypertension. She also showed generalized rigidity and involuntary movements. She was treated with fluids and iv diazepam, midazolam, clemastine, and biperiden. As the patient presented a severe insomnia and a progressive rhabdomyolysis, she was transferred to pediatric intensive care unit (ICU), where she was under treatment with cyproheptadine, mechanical ventilation, and muscular paralysis for 11 days. She was discharged from hospital a few days later with no neurological sequelae. *Conclusions*. Serotonin syndrome is still not well recognized by physicians. In our patient, the diagnosis was made early due to the history of overdose with serotonin reuptake inhibitors and the triad of mental, neurological, and autonomic signs. Parents must be educated to prevent children from having free access to drugs, avoiding self-medication or overdose.

## 1. Introduction

In the past years, the use of psychiatric drugs like serotonin reuptake inhibitors (SSRIs) has increased among children and adolescents [1]. Serotonin syndrome (SS) is a potentially life-threatening drug effect from the use of SSRIs as well as other drugs like monoamine oxidase inhibitors (MAOIs), tricycle antidepressants, over-the-counter cough medicines, antibiotics, antiemetics, drugs of abuse, and herbal products [2]. Clinical manifestations are nonspecific. It is often described as a clinical triad of mental-status changes, neuromuscular abnormalities, and autonomic dysfunction, although these symptoms may not be all present in the same patient and at the same time [2–4].

The SS in children may be misdiagnosed because it has mostly been reported in adults. However, the incidence of this condition in children has increased and it is important to recognize the clinical manifestations [2]. The authors report the case of SS in an 8-year-old child resulting in pediatric intensive care unit (ICU) admission following an overdose of SSRIs.

## 2. Case Presentation

An 8-year-old girl with behavioral problems was prescribed oral risperidone (1 mg per day) since the age of 6 and sertraline (25 mg/day) 1 month before. She was admitted in a pediatric emergency department at 4 am after she was found by her caregivers in a confusional state. Five hours before admission she had voluntarily taken 30 pills of sertraline, 1500 mg (50 mg/kg) because she had insomnia. When she arrived at the hospital, she had a marked agitation with visual hallucinations, normal pupils, diaphoresis, flushing, hypersalivation, tremor, and a strange behavior as if she was afraid of something. Her vital signs included an axillary temperature of 38.3°C, pulse of 160 beats per min (bpm), periods of hypertension (maximum of 150/96 mmHg), and polypnea with peripheral oxygen saturation of 96%. She also presented generalized rigidity with hyperreflexia and involuntary movements like myoclonus in both hands. A peripheral intravenous (iv) access was established and she was given fluids and a first dose of oral diazepam (10 mg). A venous blood gas demonstrated a pH of 7.46 with a pCO_2_ of 33 mmHg and bicarbonate of 25 mmol/L. The blood cell count was 12.03 × 10^3^/uL with 5.3% neutrophils and 26.6% lymphocytes. She had also normal renal and hepatic function with serum urea (BUN) 36 mg/dL, creatinine 0.59 mg/dL, alanine aminotransferase (ALT) 11 U/L, and aspartate aminotransferase (AST) 37 U/L. The urine toxicology excluded toxic intake. She was prescribed iv diazepam every 4 hours. Because there was no clinical improvement, she was then prescribed iv midazolam, clemastine, and biperiden. However, the clinical condition progressed with dystonic and athetotic movements of all four extremitites. The patient was not able to sleep during 48 hours and presented progressive elevation of creatine kinase (CK) at a maximum of 316 U/L. After 36 hours, the patient was sedated, submitted to muscle paralysis, and transferred to the pediatric ICU.

During the admission in ICU, due to the severity of clinical condition she was under treatment with cyproheptadine (a nonspecific serotonin antagonist) during 5 days. She was also prescribed iv midazolam, morphine, and vecuronium. For the hypertension, she was prescribed propranolol until day 10. The patient presented rhabdomyolysis with a CK maximum of 843 U/L and myoglobin of 170 *μ*g/mL at day 7, for which she was treated with fluids, furosemide, and bicarbonate perfusion until day 11. The renal and hepatic functions were always normal, but at day 8 the serum chemistry revealed an elevation of transaminases (AST 1091 U/L and ALT 452 U/L) with no signs of cholestasis. Mechanical ventilation and muscular paralysis were necessary for 11 days. After extubation, she had low muscle strength and periods of mental confusion; the vital signs were stable. She was transferred back to the pediatric department and a few days later she was discharged from hospital with a normal neurological examination, without antidepressants. At the present time, two years after this episode she is under treatment with risperidone for behavioral problems and she has no neurological sequelae.

## 3. Discussion

Serotonin (5-HT) is a neurotransmitter produced in presynaptic neurons from L-tryptophan. The concentration of serotonin available at postsynaptic receptors is regulated by a combination of feedback loops, reuptake mechanisms, and metabolism [2]. Serotonin receptors are divided into 7 types: 5-HT1 through 5-HT7. The receptors are located in the central nervous system where they are involved in behavior, mood, sleep-wakefulness cycles, muscular tone, nociception, and thermoregulation [2, 5]. The receptors located in the periphery are involved in the regulation of gastrointestinal mobility and vascular tone [2].

SS is a predictable consequence of overstimulation of serotonin receptors, although the exact pathophysiology is not very well known [4]. The incidence of SS reflects the increasing number of serotonergic agents used in pediatric and adult population [2]. The syndrome has an estimated incidence of 14 to 16% of people who overdose on SSRIs [6]. Although severe cases have been reported with an overdose of a single drug, they more often occur with a combination of two or more serotonergic drugs, even if they are used at therapeutic dose [7]. Because most cases are reported in adults and the variability of clinical manifestations, the syndrome may be misdiagnosed in children [2, 4].

SS is characterized by a triad of mental, autonomic, and neurological disorders that usually begins suddenly less than 24 hours after the beginning of treatment or overdose [2, 4, 5, 8, 9]. Mild cases present with mydriasis, diaphoresis, tachycardia, tremor, clonus, and hyperreflexia, typically more prominent in the lower extremities [5, 8]. Moderate cases may also present gastrointestinal symptoms, fever, and rhabdomyolysis. Neurological symptoms such as altered mental status, insomnia, and agitation are also common [5]. Severe cases may present with profound hypertension and tachycardia, high fever, seizures, and delirium and proceed to coma [8]. In 2003, Dunkley et al. [10] published the Hunter Serotonin Toxicity Criteria, which are more sensitive and specific than Sternbach's criteria. In the presence of a serotonergic agent, there must coexist spontaneous clonus, inducible clonus, and agitation or diaphoresis; ocular clonus and agitation or diaphoresis; tremor, and hyperreflexia; hypertonicity and temperature > 38°C; and ocular clonus or inducible clonus. Only the following variables are required for accurately predicting serotonin toxicity: clonus, agitation, diaphoresis, tremor, and hyperreflexia.

There are no laboratory tests that confirm or exclude the diagnosis of SS. To reach the diagnosis, a history of use of serotonergic agent, clinical manifestations, and the exclusion of other conditions are required [2, 5].

In patients with confusion and neurological symptoms, it is important to make the differential diagnosis with other conditions, such as the neuroleptic malignant syndrome, dystonic reactions, carcinoid syndrome, and encephalitis. There are many clinical features shared by these conditions, but clonus, hyperreflexia, and flushing are the most specific signs for SS [7, 9].

There are no guidelines or consensus for diagnosis or treatment of this syndrome. The management of patients with SS relies on early recognition, the removal of the precipitating drugs, and supportive care, including external cooling, antihypertensive drugs, muscular paralysis, and sedation [4, 8, 9]. Benzodiazepines are the most important drug in the management of the majority of cases. They have been advocated as treatment for this syndrome due to its nonspecific inhibitory effects on serotonergic transmission [4, 9, 11]. The intensity of therapy depends on the severity of illness. Severe cases with hyperthermia should receive supportive care, benzodiazepines, and immediate sedation with neuromuscular paralysis [2]. Control of hyperthermia should be attempted with benzodiazepines and muscular paralysis induced by nondepolarizing agents as vecuronium, followed by mechanical ventilation. There is no rule for antipyretic agents because the hyperthermia is due to excessive muscular activity and not to hypothalamic dysfunction. Hypertension and tachycardia should be treated with short-acting agents as nitroprusside [2]. Propranolol should be avoided because it might cause shock in patients with autonomic instability. However, our patient was treated with this drug with good response and no side effects.

Cyproheptadine is an antihistamine with non-specific antagonism properties at 5-HT1 and 5-HT2 receptors [2, 9–12]. The treatment of SS is based on experience, since clinical trials are not available [3]. Graudins reported 5 cases of SS with mild-to-moderate features that were treated with one single dose of cyproheptadine, all with good outcomes [11]. Other reports present cyproheptadine as an important drug for the management of this syndrome, especially in mild-to-moderate cases, with no adverse effects [13, 14]. Cyproheptadine is only available as an oral preparation. Pediatric dosing advice has been published with a recommended dose of 0.25 mg/kg/day, every 2 hours until symptoms improvement [2, 3, 8, 11]. In our patient, cyproheptadine was maintained for 5 days and mechanical ventilation and muscular paralysis were necessary for 11 days. In some cases, chlorpromazine has been reported to be effective in the management of serotonin syndrome [15]. However, it should be carefully managed since the drug can cause hypotension, dystonic reactions, and a neuroleptic syndrome [2, 11].

In conclusion, SS is still not well recognized by physicians. In our patient, the diagnosis was made early due to the history of overdose with SSRIs and the triad of mental, neurological, and autonomic signs. Cessation of serotonergic medication and supportive care remain the mainstay of therapy. Muscular paralysis and mechanical ventilation may be necessary to prevent renal and hepatic failure due to rhabdomyolysis. Cyproheptadine should be considered in mild-to-severe cases, although patients' outcome is still not known. Parents must be educated to prevent children from having free access to drugs, avoiding self-medication or overdose.
